# Dental Anxiety and Its Association with Dietary Intake and Food Groups: A Cross-Sectional Study

**DOI:** 10.3390/dj11100240

**Published:** 2023-10-17

**Authors:** Lina Begdache, Eeshah Ahmed, Sana Malik, Muhammet Furkan Karakaya

**Affiliations:** 1 Health and Wellness Studies Department, Binghamton University, Binghamton, NY 13902, USA; 2 School of Dentistry, University of the Pacific Arthur A. Dugoni, San Francisco, CA 94103, USA; e_ahmed1@u.pacific.edu; 3 School of Dental Medicine, University at Buffalo, Buffalo, NY 14260, USA; ssmalik2@buffalo.edu; 4 Political Science Department, Binghamton University, Binghamton, NY 13902, USA; mkaraka1@binghamton.edu

**Keywords:** dental anxiety, mental health, age, nutrition, western diet, Mediterranean diet

## Abstract

Although there is an established connection between diet and mental health, the relationship between diet and dental anxiety has not been examined yet. The purpose of this study was to fill this gap by assessing the association between diet quality, mental distress, and dental anxiety. The data was collected through an anonymous Google Forms survey. The survey consisted of a modified version of the validated Food–Mood Questionnaire and the Modified Dental Anxiety Scale with questions about demographics, dental health, and dental health anxieties. Data collection was performed over seven months, from April to October 2021. Data were analyzed using Pearson’s correlation coefficient in SPSS version 25.0 and STATA 17 for sample size calculation, data processing and regression analyses. A total of 506 responses were collected. Our data verified that diet quality modulates dental anxiety. Women exhibited a stronger link with dental anxiety than men (*p* < 0.01). Consumption of sugary foods was associated with different attributes of dental anxiety (*p* < 0.01). Low-quality energy-dense foods and dairy were associated with dental anxiety, whereas caffeine, meat, nuts, and green leafy vegetables produced a negative correlation. This cross-sectional study provides proof of concept that dietary patterns are potentially associated with dental anxiety.

## 1. Introduction

### 1.1. Dental Anxiety, Oral Health, and Mental Health

Dental anxiety refers to a high degree of fear, anxiety, or phobia associated with dental procedures due to negative experiences at dental offices [1]. Dental anxieties are not only experienced at the time of the dental appointment but also outside the dental office environment.

Anxiety from dental treatments could lead to physiological adverse effects such as increased heart palpitation and rapid breathing [2], creating a state of apprehension.

Irrational fear and avoidance of dental treatment due to severe anxiety could lead to dental phobia [1]. Consequently, the constant fear eventually impacts oral health and increases the risk of dental-related infections [3]. This is of utmost concern for the under-resourced communities that lack effective medical and dental treatments. Ethnic and racial minorities, the elderly, and populations from rural areas and of low socio–economic status are considered vulnerable populations that are more likely to report poor oral health status [4]. Low diet quality has an impact on oral health as well. Cariogenic diets, typically high in sugar, raise the risk of oral infectious diseases [5].

Several reports describe the associations between mental health and dental-related anxieties [3,4,6,7]. In fact, those diagnosed with mental health illnesses are at a higher risk of developing oral health complications [4]. They are more likely to encounter poor oral health conditions and are unlikely to address their dental needs.

### 1.2. Nutrition, Mental Health, and Dental Anxiety

The relationship between nutrition and mental health is becoming evident, as poor nutrition negatively affects mental health. A high-nutrient-dense diet fuels the human body [8] and plays a pivotal role in brain structure and chemistry. However, food that is high in simple carbohydrates is typically devoid of several beneficial nutrients. On the other hand, complex carbohydrates are rich in fiber and several micronutrients, which were reported to promote mental well-being. High-fiber and low-glycemic-index foods have a positive effect on mood via the stabilization of blood sugar [9]. Caffeine is a central nervous system stimulant that is commonly found in coffee, tea, and energy drinks, among others. Simple sugars and caffeinated beverages trigger fluctuations in mood, anxiety, and depression [6,9,10]. Additionally, deficiencies in essential amino acids and healthy fats such as omega-3 fatty acids can severely impact brain function. Micronutrients that are typically found in a high-quality diet and modulate mood include B vitamins, vitamin D, vitamin C, and magnesium, among others. Hence, a low-quality diet may impact mood through homeostatic disturbances linked to nutrient deficiencies.

Patients with poor oral health, especially those with missing teeth, may consume fewer servings of high-nutrient foods such as fruits, vegetables, and meat because these foods are difficult to masticate [6]. This hurdle affects the way patients cook their meals, leading them to resort to fast foods or prepackaged food items, increasing the risk of mental distress. Eventually, this cyclic event worsens oral and mental health.

Caffeine has a dual impact on mood. Caffeine boosts mental performance by reducing sensations of fatigue and promoting dopamine release, increasing the sense of well-being. Contrarily, caffeine stimulates the hypothalamic-pituitary-adrenal (HPA) axis in a dose-dependent manner, which increases the risk of mental distress. However, its impact on dental anxiety remains unknown. According to Verster et al., daily caffeine consumption of 170 mg/day is highest among 35- to 49-year-old individuals [11], with men consuming greater amounts than women. As expected, most of the caffeine consumption comes from coffee.

Nutritional status and the risk of oral diseases exhibit a reciprocal relationship, which may influence the level of dental anxiety sensed at dental offices. Patients consuming a high-quality diet experience stable mental health, which results in reduced mood fluctuations, dental-related anxieties, and phobias [6]. Although several published reports explain the effects of nutrition on oral health, the relationship between nutrition and dental-related anxieties is still unknown.

Combining all the evidence, diet quality has a potential modulatory effect on mental health, which may impact dental anxiety. However, no report in the literature describes the effect of diet on dental anxiety. Therefore, the purpose of this study was to fill this gap and test this proof of concept. The null hypothesis is that diet quality has no relationship with dental anxiety. Another purpose was to assess the impact of diet quality and food groups on anxieties related to visiting the dentist and receiving specific dental treatments.

## 2. Materials and Methods

### 2.1. Participants and Study Design

The study protocol STUDY00002931 was reviewed and approved by the Institutional Review Board at Binghamton University on 8 April 2021. A built-in consent form was included in the survey, and described the study purpose, procedure, risk, benefits, and contact name and number of the principal investigator. The inclusion criteria were adults 18 years of age or older who could read and understand English. Participation in the study was purely voluntary, and no compensation was provided. The anonymous questionnaire was administered online, in dental offices, and through social media platforms. Dental offices from different geographical locations were selected to enhance the representation of the population. The rationale behind using two different methods of data collection is to broaden the types of responses and improve the validity of the results.

A QR code was provided at several dental offices for patients to access the survey using their electronic devices on-site. No patient history was examined before conducting this study. Data collection was performed for seven months, from April to October 2021.

### 2.2. Sample Size Calculations

To produce statistical significance, the sample size was calculated using Powerlog function of STATA software 17.0 (StataCorp LLC, Lakeway Drive, College Station, TX, USA). STATA has a function to measure the power of logistic regression analysis to determine the minimum number of observations for survey analysis. The Powerlog function requires effect size values and an alpha value to calculate the minimum number of observations for the logistic regression analysis. The alpha value is 0.05 for a one-tailed test, and it is 0.25 for a two-tailed test. The effect size is “the probability that the response variable equals 1 when the predictor is at the mean”, which is denoted as p1 value in STATA language, and “the probability that the response variable equals 1 when the predictor is one standard deviation above the mean”, which is denoted as p2 value. Powerlog generated 154 as the minimum number of observations for the 0.80 power (conventional level of power) and 260 for the 0.90 power level for the one-tailed test after inputting the values from our model. Also, we received 196 and 260 for the 0.80 and 0.90 power levels, respectively, when we set the alpha values at 0.025 for the two-tailed test. Our study had 506 respondents, indicating that the sample size fulfilled the minimum number of observations at both the 0.80 and 0.90 power levels.

### 2.3. Data Collection and Surveys

A modified version of the Food–Mood Questionnaire (FMQ) was used for dietary and mood data collection. FMQ is a validated and reliable tool that assesses dietary patterns and frequency of food groups such as whole grains, meat/chicken/turkey, fish, green leafy vegetables, fruits, dairy products, caffeine, beans, seafood, fast foods, supplement use such as fish oil and multivitamins, high-glycemic-index foods, and nuts [12]. The frequency of fast food, pre-packaged meals, and sugary foods consumed was also assessed (Table 1). Dietary patterns were categorized as Western, Asian, Mediterranean, Korean, Eastern, Hindu, South American, or Other. Mental distress within FMQ was evaluated using the Kessler psychological distress scale (K-6), which assesses mental distress, with four potential answers: “None of the time”; “A little of the time”; “Some of the time”; “Most of the time”; and “All of the time”, based on the Kessler-6 (K6) scale, [13]. The six different categories are: feeling nervous, depressed, worthless, restless/fidgety, hopeless, and feeling everything was an effort. The sum of the total scores was used to describe the level of mental distress, with variables ranging between 0 and 24. Answers range from none to five times or more. Demographic questions included gender, age groups, race, zip code of residence, the highest education achieved, and employment status.

A modified version of the Dental Anxiety Scale (MDAS) was used for oral health and dental anxiety data collection [14], which included questions on anxiety levels when visiting the dentist, the next day, sitting in the waiting room for treatment, having a tooth drilled, scaled, polished, and receiving a local anesthetic injection in the gum (Table 1). The survey also included questions on self-perceived oral health status, dental health insurance, duration since the last visit to the dentist, and fear of having a tooth extracted.

### 2.4. Data Analysis

**Figure 1:**
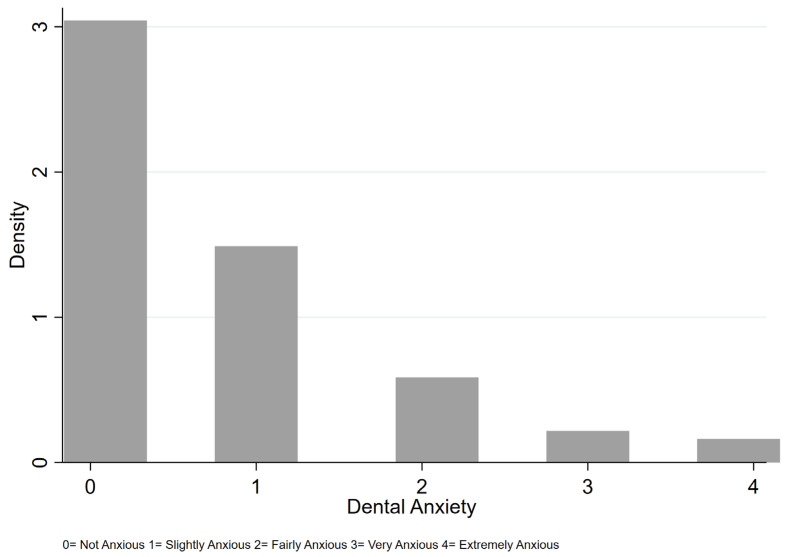
The different degrees of dental anxiety in the sample.

For the regression analysis models, the dependent variable, dental anxiety, was obtained from a five-point scale interview question and was converted from a multinomial categorical into a binary variable, where 0 represents people who reported ‘not anxious’ while 1 represents people who reported any level of anxiety: ‘slightly anxious’, ‘fairly anxious’, ‘very anxious’, or ‘extremely anxious’. The variable comes from the survey question that we ask: “how do you feel if you have a dental appointment tomorrow?” The purpose of this conversion was to improve the goodness of fit of the logistic regression model with the given data type and its distribution. In addition to the main independent variable, dietary patterns, gender, age, race, and education were controlled for. Gender is a binary variable, with 0 representing females and 1 representing males in the sample. The education variable is categorical and evaluates the highest education level completed. The race variable is a nominal variable with seven categories: American Indian or Alaska Native, Asian, African American, Hispanic, Native Hawaiian or Pacific Islander, White, and other. Age categories included 18–29, 30–39, 40–49, and 50 and above. The hypothesis is that different age categories are differentially correlated with dental anxiety. Since the categorical version of the dental anxiety variable is skewed (Figure 1), which can generate a bias, a binary version was used to obtain a more accurate measurement of dental anxiety. Data were analyzed using STATA 17 for sample size calculation, data processing and regression analyses, and SPSS 25.0. (IBM, SPSS Inc., Chicago, IL, USA) for Pearson’s correlation coefficient analysis.

## 3. Results

Demographics

Out of 506 survey responses, 66.4% of respondents were female, 41.6% were between 18 and 29 years of age, 54% were white, 35.6% had completed 2 or 4 years of a college degree, and 63.8% were employed. In addition, 43.1% of participants perceived their oral health status as good, 28.3% perceived themselves as having excellent oral health, and the remaining 23.7% and 4.9% perceived themselves as having average and poor oral health, respectively. About 83% of participants were covered by dental health insurance, while 17.3% were not. Close to 44% of participants had visited the dentist within the last 6 months, 29.6% in the last 6–12 months, 19.4% in the last 1–2 years, and 7.3% over 2 years ago (Table 2).

Two logistic regression models were used to test the hypothesis that diet quality may be associated with dental anxiety. Table 3 illustrates the results from the logistic regression analyses. Model 1 assesses the effects of demographics on dental anxiety, while Model 2 examines the effects of demographics and dietary patterns on dental anxiety. The results from the two models suggest that diet has a positive and statistically significant association with dental anxiety at *p* < 0.05 level. Moreover, gender is statistically significant at *p* < 0.01 level and has a negative correlation with dental anxiety. Particularly, being a female compared to being a male increases the likelihood of having dental anxiety. In other words, females are relatively more prone to experiencing dental anxiety after controlling for confounding factors.

Education and race do not have a statistically significant impact on dental anxiety after controlling for dietary patterns and other confounding variables. Excluding the effect of dietary patterns, individuals between 18 and 29 years old and 50 and 59 years old are relatively less likely to have dental anxiety (Model 1, Table 3). Interestingly, when dietary patterns were factored in, only the young age group (18–29 years) had a significant inverse association with dental anxiety (Model 2, Table 3). Thus, the results from Table 3 strongly suggest that dietary patterns have a significant effect on dental anxiety.

**Figure 2:**
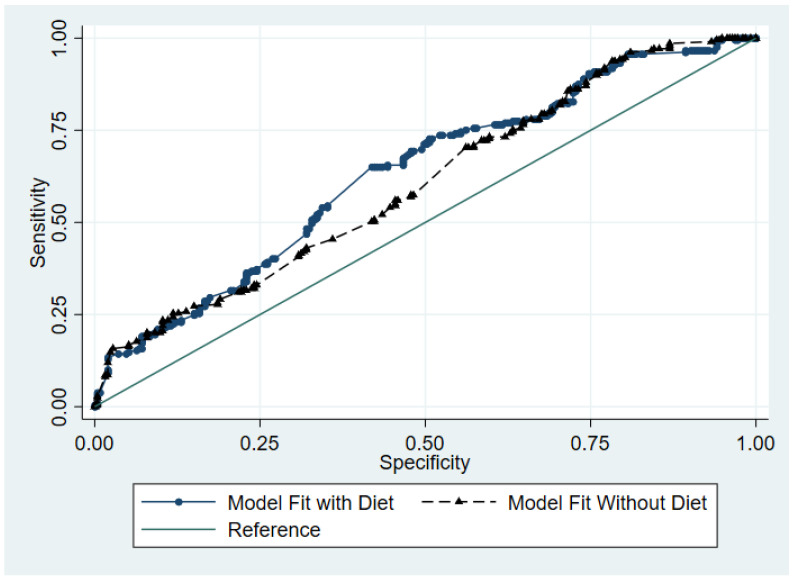
ROC Curves for Models 1 and 2. Effects of Diet Types on Dental Anxiety.

However, to better appreciate the power gained through the inclusion of dietary patterns in Model 2, a receiver operating characteristic (ROC) test was performed to compare both models’ fit (Figure 2). The ROC test helps to evaluate the performance of a binary logistic regression model based on the true positive rate against the false positive. In this figure, the top line represents Model 2’s performance, while the lower one represents that of Model 1. Model 2 classifies approximately 0.633 areas out of 1.000 under the 45-degree line, while model 1 classifies 0.600 areas out of 1.000. Therefore, compared to model 1, model 2 provides a better fit, which suggests that dietary patterns better explain dental anxiety than demographics alone. In other words, we can confidently reject the null hypothesis, which states the areas under the ROC of the two models are the same.

In sum, the combination of Table 3 and Figure 2 results confirms that diet has a statistically significant impact on dental anxiety. Particularly, Figure 2 bolsters our findings on the effects of diet on having dental anxiety and indicates that adding the dietary pattern variable into the model provides a more accurate explanation for having dental anxiety.

The next step was to investigate the most prominent dietary pattern associated with dental anxiety. For that purpose, four additional models were run to explore the effects of the Western, Mediterranean, and Asian diets on dental anxiety. The rationale behind using multiple models was to check the robustness of our findings with different model specifications and explore the differential effects of certain diet types on dental anxiety. To prevent multicollinearity effects, the diet variable was dropped from all models. Model 3 examined the effects of the Western diet on the likelihood of having dental anxiety. The results from Model 3 indicate that the Western diet has a statistically significant and positive association with dental anxiety. Therefore, the Western diet increases the likelihood of having dental anxiety even after controlling for other demographic factors at *p* < 0.1. On the other hand, model 4 shows that the Mediterranean diet has a negative and statistically significant impact on dental anxiety at *p* < 0.1 level, which suggests that the Mediterranean diet decreases the likelihood of having dental anxiety. The results from model 5 indicate that the Asian diet has no statistically significant correlation with dental anxiety when controlling for demographic variables. Model 6 shows that the effects of three diets combined on dental anxiety have no statistically significant impact on dental anxiety, suggesting that a mix of various diet types is not associated with dental anxiety (Table 4).

Next, we aimed to investigate the effects of different food groups on dental anxiety to better understand the effect of diet quality. All food variables were treated as binary, reflecting consumption or no consumption of food. The results from Table 5 indicate that sugary foods have a positive and statistically significant impact on dental anxiety with different model specifications, which means that consumption of sugary foods increases the likelihood of having dental anxiety. These findings were significant in different model specifications.

Likewise, dairy products have a positive and statistically significant correlation with dental anxiety. On the other hand, coffee and meat (red and white) have a statistically negative association with dental anxiety, which means that they may decrease the likelihood of having dental anxiety. The results are robust with different model specifications. Lastly, the results from Table 5 indicate that high-glycemic-index foods such as rice and pasta, fruit, nuts, vegetables, and fast food have no statistically significant impact on dental anxiety.

### 3.1. Dietary Behavior Correlated with General Anxiety

The following stage was to assess the association between food groups and specific mental distress characteristics and dental anxiety. There is a statistically significant positive correlation between the consumption of sugary foods and feeling restless/fidgety, nervous, hopeless, worthless, and depressed (*p* < 0.01). Several statistically significant positive correlations emerged between the consumption of pre-made/fast foods and feeling restless/fidgety, nervous, hopeless, worthless, and depressed (*p* < 0.01). Interestingly, no statistically significant correlations were found between the consumption of pre-made/fast foods and dental-specific anxieties (Table 6).

### 3.2. Dental Anxiety, Gender, and Sugary Foods, as Well as Caffeine Consumption

Females are more likely to experience dental anxiety if they are going to the dentist tomorrow, sitting in the waiting room waiting for treatment, or undergoing various dental procedures, including having a tooth drilled, having a tooth scaled and polished, receiving a local anesthetic injection, and having a tooth pulled out. There were statistically significant positive correlations between the consumption of sugary foods and various dental anxieties, including anxiety felt if going to the dentist tomorrow, sitting in the waiting room for treatment, having a tooth drilled, having a tooth scaled and polished, receiving a local anesthetic injection, and having a tooth pulled out (*p* < 0.01) (Table 7).

### 3.3. Dietary Patterns and Dental-Related Anxiety

There were statistically significant positive correlations between frequent consumption of fruits and consumption of nuts/flaxseed, dark green leafy vegetables, and supplement use (*p* < 0.01), reflecting a healthy dietary pattern and habits. Statistically significant negative correlations were detected between frequent consumption of fruits and ingesting high-glycemic-index foods as well as pre-made/pre-packaged foods (*p* < 0.01), suggesting that those who follow a healthy dietary pattern are less likely to make unhealthy food choices. Statistically significant negative correlations were found between dental anxiety about having a tooth pulled out and consumption of nuts/flaxseed (*p* < 0.01) and high-quality proteins such as red meat, chicken, turkey, and fish (*p* < 0.01). Dental anxiety regarding having a tooth scaled and polished was negatively correlated with frequent consumption of leafy green vegetables (*p* < 0.05). A statistically significant negative correlation emerged between consuming the Western diet and dental anxiety related to having a tooth drilled (*p* < 0.01). Another statistically significant negative correlation was detected between consuming the Western diet and dental anxiety related to having a tooth scaled and polished (*p* < 0.05) (Table 8).

## 4. Discussion

The purpose of this study was to test the hypothesis that diet quality is associated with dental anxiety, which fills a gap in the literature. Another purpose was to assess the impact of nutrition on mental health, with a specific focus on dental anxiety. The first step was to examine the effects of dietary patterns on the likelihood of dental anxiety among individuals. Several interesting findings were revealed. Gender, age, nutrition, education, and employment play a significant role in dental anxiety. Most patients in our cohort experienced dental anxiety, with a higher occurrence in females and young adults. Heightened anxiety in females may be explained by hormonal repertoires such as corticosterone, estrogen, and the human corticotropin-releasing factor, which all facilitate anxiety-related symptoms and disorders [15]. The limbic system, which comprises the hippocampus and amygdala, causes elevated emotions such as negative feelings. Females appear to have a stronger activation of the left central amygdala (CE), which is associated with anxiety [16]. Another interesting finding is that patients with lower employment and education status were more likely to indicate generalized anxiety disorders in addition to dental anxiety. Younger adults also exhibited a higher level of anxiety, which confirms previously published reports [17,18]. This finding could be related to the incomplete maturity of the prefrontal cortex, the part of the brain that rationalizes thoughts and controls emotions.

### 4.1. Dental Anxieties and Food Quality

Participants who consumed a low-quality diet consisting of sugary foods, pre-packaged foods, or fast foods experienced higher levels of anxiety and depressive symptoms. These foods are typically devoid of nutrients that support brain structure and chemistry. In addition, these foods promote oxidation and inflammation, which lead to a state of oxidative stress. The lack of antioxidants due to low consumption of fruits and vegetables damages the neural network, so effective communication between different parts of the brain is disturbed. These results were further confirmed using the regression models in this study. They are in line with a previously published report that described the positive association between low-quality food intake, lower fruit and vegetable consumption, and depressive symptoms, specifically among female adult participants [19]. In addition, the Western diet, consisting of low-quality food, is typically linked with lower levels of brain-derived neurotrophic factor (BDNF), which plays a crucial role in neurogenesis and neuroplasticity [10]. Neurogenesis is vital for preserving brain volume, while neuroplasticity is important for maintaining optimal neural connections.

Vegetable consumption could mitigate anxious and depressive symptoms by providing several minerals, vitamins, fibers, bioactive compounds, and antioxidants that are beneficial for brain health [20]. Many of these phytonutrients regulate gene expression responsible for brain homeostasis.

Additionally, the Western diet leads to gut dysbiosis, another trigger of anxiety and depressive symptoms [21]. Gut dysbiosis has been linked with dysregulation of the immune system and disruptions in neuronal and synaptic activities, leading to inflammation and poor communication among different regions of the brain, respectively. Therefore, our results align with previous research and suggest that low-quality food exacerbates the risk of mental illnesses, potentially through these physiological disturbances.

Individuals who consume dark green leafy vegetables, nuts/flaxseed, and high-quality proteins such as red meat, chicken, turkey, and fish experience lower levels of dental anxiety and fewer symptoms of anxiety and depression. The consumption of fruits was not directly correlated with either general or dental-related anxieties. Instead, statistically significant correlations were found between the consumption of fruits and nutrient-dense foods such as nuts/flaxseed, dark leafy green vegetables, and supplements. The Mediterranean diet, rich in a spectrum of nutrient-dense foods, such as whole grains, fruits, and vegetables, is associated with mental well-being and enhanced resilience [22,23]. It is believed that these nutrients exert a synergistic effect on brain function that culminates in an improved mental state. These nutrients work at the molecular level to modulate gene expression related to brain chemistry, brain health, and homeostasis.

### 4.2. Dental Anxieties and Caffeine Consumption

All the regression models generated suggest that caffeine is inversely linked to dental anxiety. The correlation study linked a higher frequency of caffeine consumption (4 cups or more a week) to lower dental anxiety. Thus, the frequency of caffeine consumption and dental anxiety are worth further investigation. Caffeine acts as a sword with a double edge. At low concentrations, caffeine boosts mood, while high levels may exacerbate the stress response [24]. Caffeine consumption is associated with physiological alterations including heart palpitations, increased anxiety, nervousness, tremors, and nausea [25]. However, caffeine boosts dopamine levels and increases brain stimulation, alertness, and euphoria [24,26]. Our results suggest that caffeine consumption between 0 and 3 times per week is correlated with higher dental anxiety, while consumption of 4 times or more per week correlates with lower dental anxiety. These results were surprising as they contradict several reports on caffeine and mental distress [25,27,28], but they may be explained using scientific evidence as well.

Caffeine is an adenosine (A_1_ and A_2a_) receptor activity antagonist, whose activation is associated with glutamate release [29] under chronic stress. Adenosine receptor genes, commonly known as A_1_ R, A_2a_ R, or ADORA_2a_, produce modulatory effects on mood [30]. Kaster et al. eloquently described the mechanism by which high caffeine consumption attenuates maladaptive responses to chronic stress. The latter increases the risk of mental health ailments due to the associated neurochemical and morphological changes in the brain [31]. Under chronic unpredictable stress (CUS), caffeine, as an adenosine receptor antagonist, offers neuroprotection against stress-induced aberrant neuroplasticity, which may explain our findings that high-caffeine consumers may be undergoing neuroadaptive responses during stressful experiences. In addition, A_2a_R polymorphisms are associated with fear and anxiety attacks [24]. Those with A_2a_ genotype polymorphisms are susceptible to higher anxiety after caffeine consumption and are less likely to consume caffeine regularly. Therefore, regular caffeine consumption is less likely to induce dental anxiety as a beneficial adaptive response may have developed, whereas sporadic caffeine intake may exacerbate dental anxiety as individuals are more vulnerable to the effects of caffeine.

Rogers et al. investigated the anxiogenic effects of caffeine on habitual caffeine consumers and sporadic caffeine consumers [30]. Infrequent caffeine consumers tend to carry TT genotypes, whereas frequent caffeine consumers tend to have CC or CT genotypes. Hence, infrequent caffeine consumers with TT genotypes experience greater anxiety, while frequent caffeine consumers with CC or CT genotypes feel little to no change in anxiety level. When habitual caffeine consumers were presented with caffeine after being weaned off, their anxiety levels soared. In contrast, consumers who maintained a certain caffeine dosage felt no change in anxiety level when presented with caffeine. While the relationship between caffeine consumption and anxiety is explained using existing literature on a genetic basis, other studies examined caffeine tolerance patterns. A lower frequency of caffeine consumption may suggest sporadic intake, while a higher frequency assumes consistent intake. Low-caffeine consumers (less than 500 mg caffeine per week) felt more anxious when given caffeine, whereas fewer anxiogenic effects were felt among high caffeine consumers (minimum 400 mg caffeine per day) [23]. These findings suggest that habitual caffeine consumers build a tolerance to caffeine and its anxiogenic effect as opposed to sporadic caffeine consumers. Combining all the evidence, adenosine receptor antagonism coupled with beneficial caffeine-induced adaptation may reduce anxiolytic symptoms associated with dental anxiety such as tooth extraction, polishing, and drilling [32,33]. However, further research is needed to confirm this theory.

### 4.3. Limitations and Future Direction

Our study has several strengths and limitations. The large sample size and the filling of a gap in the literature are its strengths. The regression models, as well as testing the robustness of the models, are also strengths. The study followed a cross-sectional design, which means these results need to be interpreted with caution. The fact that the study did not assess exercise, as a potential modulator of mental health, is a limitation. Physical activity, of at least 20 min, 3 or 4 times per week, has a significant positive impact on mental well-being [34]. Moreover, this study did not record breakfast consumption. A significant relationship between skipping breakfast and physiological disorders of anxiety and depression has been reported among adults [35]. For future direction, the type and duration of exercise must be assessed in relation to dental anxiety. Caffeine intake must also be further quantified and evaluated. This study examined the frequency of consumption per week but not the varying caffeine content between beverages.

## 5. Conclusions

Our findings propose that diet quality, gender, and age impact dental anxiety. Sugary foods and fast food were also associated with dental anxiety, while higher-quality food was generally associated with mental wellbeing. The bidirectional relationships between oral health, mental health, and nutrition are topics to be further analyzed to validate the findings of this study. Future works should explore the connection between exercise, breakfast consumption, and dental anxiety. Whilst the association between dentistry and dietetics has not been explored previously, this study acts as a bridge connecting nutrition with dental anxiety.

## Figures and Tables

**Table 1:** MDAS, Modified FMQ, and Kessler-6 questions.

MDAS
If you were sitting in the waiting room, how would you feel?
If you were about to have a tooth drilled, how would you feel?
If you were about to have your teeth scaled and polished, how would you feel?
If you were about to have a local anesthetic injection in your gum, how would you feel?
**Modified FMQ**
On an average week, how many times do you consume coffee or other sources of caffeine?
On an average week, how many times do you consume fruits?
On an average week, how many times do you consume nuts, including flaxseed?
On an average week, how many times do you consume rice and/or pasta?
On an average week, how many times do you consume red meat, chicken, turkey, or fish?
On an average week, how many times do you consume dark green leafy vegetables?
On an average week, how many times do you consume fast foods and/or pre-made or packaged food?
On an average week, how many times did you consume sugary foods (candy, chocolate, or sweets)?
On an average week, how many times do you take supplements? (multivitamins, fish oil…)
**Kessler-6**
During the past 30 days, about how often did you feel hopeless?
During the past 30 days, about how often did you feel restless or fidgety?
During the past 30 days, about how often did you feel so depressed that nothing could cheer you up?
During the past 30 days, about how often did you feel that everything was an effort?
During the past 30 days, about how often did you feel worthless?

**Table 2:** Demographic Representation and Oral Health Information.

Independent Variables				
Biological Sex	*Women*	*Men*		
	336	170		
**Age**	18–29	50–59		
	210	72		
	30–39	60–69		
	94	46		
	40–49	70 *or Older*		
	62	21		
**Race**	*White*	*Native Hawaiian or Pacific Islander*		
	273	2		
	*Black or* *African-American*	*Hispanic or Latino*		
	29	30		
	*Asian*	*Other*		
	124	44		
**Education**	*Less than High School*	*Master’s Degree*		
	5	61		
	*High School*	*Doctoral Degree*		
	176	29		
	2 *or* 4 *Years of* *College Degree*	*Professional Degree*		
	180	55		
**Employment Status**	*Employed*	*Other* (*Homemaker*, *student*, *retiree*)		
	323	131		
	*Unemployed*			
	52			
**Self-perceived oral health status**	*Excellent*	*Good*	*Average*	*Poor*
	143	218	120	25
**Dental Insurance Coverage**	*Yes*	*No*		
	417	87		
**Duration since last visit to the Dentist**	*Within* 6 *Months*	6–12 *Months*	1–2 *Years*	*More than* 2 *Years*
	220	149	98	37

**Table 3:** Effects of Diet on Dental Anxiety with Demographics.

	Model 1	Model 2
Variables	Dental Anxiety	Dental Anxiety
Diet		0.0382 **
		(0.0186)
Gender	−0.790 ***	−0.775 ***
	(0.206)	(0.214)
Education	−0.0861	−0.0567
	(0.0579)	(0.0603)
Race	−0.0199	−0.0700
	(0.0432)	(0.0534)
Age 18–29	−0.558 *	−0.609 *
	(0.297)	(0.312)
Age 30–39	0.355	0.208
	(0.332)	(0.352)
Age 40–49	−0.499	−0.473
	(0.369)	(0.389)
Age 50–59	−0.623 *	−0.470
	(0.357)	(0.384)
Constant	1.495 ***	1.164 **
	(0.509)	(0.545)
Observations	505	462

Standard errors in parentheses *** *p* < 0.01, ** *p* < 0.05, and * *p* < 0.1.

**Table 4:** Effects of Different Diet Types on Dental Anxiety with Demographics.

	Model 3	Model 4	Model 5	Model 6
Variables	Dental Anxiety	Dental Anxiety	Dental Anxiety	Dental Anxiety
Gender	−0.787 ***	−0.788 ***	−0.759 ***	−0.783 ***
	(0.214)	(0.214)	(0.213)	(0.215)
Education	−0.0576	−0.0608	−0.0743	−0.0658
	(0.0603)	(0.0601)	(0.0600)	(0.0609)
Race	−0.0584	−0.0109	−0.0377	−0.0547
	(0.0522)	(0.0442)	(0.0479)	(0.0546)
Age 18–29	−0.584 *	−0.545 *	−0.585 *	−0.580 *
	(0.312)	(0.311)	(0.311)	(0.313)
Age 30–39	0.249	0.349	0.235	0.263
	(0.351)	(0.351)	(0.352)	(0.356)
Age 40–49	−0.433	−0.352	−0.503	−0.410
	(0.388)	(0.392)	(0.390)	(0.395)
Age 50–59	−0.428	−0.371	−0.477	−0.419
	(0.383)	(0.385)	(0.384)	(0.387)
Western Diet	0.423 *			0.114
	(0.236)			(0.288)
Mediterranean Diet		−0.601 *		−0.591
		(0.327)		(0.385)
Asian Diet			−0.530	−0.561
			(0.351)	(0.381)
Constant	1.386 ***	1.414 ***	1.613 ***	1.680 ***
	(0.537)	(0.538)	(0.561)	(0.570)
Observations	462	462	462	462

Standard errors in parentheses *** *p* < 0.01, and * *p* < 0.1.

**Table 5:** Effects of Different Types of Foods on Dental Anxiety.

	Model 7	Model 8	Model 9	Model 10
Variables	Dental Anxiety	Dental Anxiety	Dental Anxiety	Dental Anxiety
Sugary food	1.072 ***	1.121 ***	1.200 ***	1.207 ***
	(0.413)	(0.429)	(0.436)	(0.455)
Dairy		1.532 **	1.486 **	1.477 **
		(0.728)	(0.726)	(0.729)
Coffee (caffeine)		−0.633 *	−0.632 *	−0.649 *
		(0.381)	(0.380)	(0.382)
Meat		−0.930 **	−0.876 *	−0.872 *
		(0.470)	(0.477)	(0.479)
Rice & pasta			−0.579	−0.561
			(0.387)	(0.396)
Fruit				−0.286
				(0.669)
Nuts				0.0399
				(0.205)
Vegetables				0.590
				(0.788)
Fast food				0.0139
				(0.268)
Constant	−1.216 ***	−1.281	−0.820	−1.165
	(0.403)	(0.814)	(0.865)	(1.178)
Observations	507	507	507	507

Standard errors in parentheses *** *p* < 0.01, ** *p* < 0.05, and * *p* < 0.

**Table 6:** Dietary Factors and General Anxieties.

Food Groups	Symptoms	Correlation
Sugary Food	Restless and Fidgety	0.192 **
Sugary Food	Nervous	0.229 **
Sugary Food	Hopeless	0.162 **
Sugary Food	Nothing cheers you up	0.125 **
Sugary Food	Everything was an effort	0.186 **
Sugary Food	Worthless	0.174 **
Fast Food	Restless and Fidgety	0.116 **
Fast Food	Nervous	0.105 **
Fast Food	Hopeless	0.167 **
Fast Food	Nothing cheers you up	0.161 **
Fast Food	Everything was an effort	0.116 **
Fast Food	Worthless	0.141 **

** *p* < 0.05.

**Table 7:** Correlations between Dental Anxiety Level and Sugary Foods.

Dental Anxiety Level	Female	Consumption of Sugary Food
Going to the dentist tomorrow	−0.171 **	0.178 **
Sitting in the waiting room	−0.195 **	0.220 **
Tooth Drilled	−0.259 **	0.175 **
Tooth pulled out	−0.118 **	0.175 **
Anesthetic injection in your gum	−0.239 **	0.225 **

** *p* < 0.05.

**Table 8:** Correlation between Food Groups and Dental-Related Anxieties.

Food Groups	Food Groups	Correlation
Fruits	Nuts	0.322 **
Fruits	High-Glycemic-Index Food	−0.138 **
Fruits	Dark green leafy vegetables	0.415 **
Fruits	Fast food	−0.272 **
Fruits	Multivitamin supplements	0.223 **
**Dietary Pattern**	**Dental Related Anxiety**	**Correlation**
Fruits	Tooth pulled out	−0.115 **
Dark green leafy vegetables	Tooth pulled out	−0.106 *
Meat	Tooth pulled out	−0.102 *
Western Diet	Tooth pulled out	−0.146 **
Western Diet	Tooth scaled and polished	−0.111 *

** *p* < 0.05 and * *p* < 0.1.

## Data Availability

Data is available upon request.
